# Association of Subclinical Hypothyroidism and Cardiovascular Disease With Mortality

**DOI:** 10.1001/jamanetworkopen.2019.20745

**Published:** 2020-02-07

**Authors:** Kosuke Inoue, Beate Ritz, Gregory A. Brent, Ramin Ebrahimi, Connie M. Rhee, Angela M. Leung

**Affiliations:** 1 Fielding School of Public Health, Department of Epidemiology, University of California, Los Angeles; 2 David Geffen School of Medicine, Department of Neurology, University of California, Los Angeles; 3 David Geffen School of Medicine, Division of Endocrinology, Diabetes, and Metabolism, Department of Medicine, University of California, Los Angeles; 4 VA Greater Los Angeles Healthcare System, Los Angeles, California; 5 David Geffen School of Medicine, Division of Cardiology, Department of Medicine, University of California, Los Angeles; 6 Harold Simmons Center for Chronic Disease Research and Epidemiology, University of California Irvine School of Medicine, Orange

## Abstract

**Importance:**

Subclinical hypothyroidism is a common clinical entity among US adults associated in some studies with an increase in the risk of cardiovascular disease (CVD) and mortality. However, the extent to which CVD mediates the association between elevated serum thyrotropin (TSH) and mortality has not yet been well established or sufficiently quantified.

**Objective:**

To elucidate the extent to which subclinical hypothyroidism, elevated serum TSH and normal serum free thyroxine, or high-normal TSH concentrations (ie, upper normative–range TSH concentrations) are associated with mortality through CVD among US adults.

**Design, Setting, and Participants:**

This cohort study relied on representative samples of US adults enrolled in the National Health and Nutrition Examination Survey in 2001 to 2002, 2007 to 2008, 2009 to 2010, and 2011 to 2012 and their mortality data through 2015. Data were analyzed from January to August 2019.

**Main Outcomes and Measures:**

Cox proportional hazards regression models were used to investigate associations between the TSH concentration category (subclinical hypothyroidism or tertiles of serum TSH concentrations within the reference range; low-normal TSH, 0.34-1.19 mIU/L; middle-normal TSH, 1.20-1.95 mIU/L; and high-normal TSH, 1.96-5.60 mIU/L) and all-cause mortality. Mediation analysis was used within the counterfactual framework to estimate natural direct associations (not through CVD) and indirect associations (through CVD).

**Results:**

Of 9020 participants, 4658 (51.6%) were men; the mean (SD) age was 49.4 (17.8) years. Throughout follow-up (median [interquartile range], 7.3 [5.4-8.3] years), serum thyroid function test results consistent with subclinical hypothyroidism and high-normal TSH concentrations were both associated with increased all-cause mortality (subclinical hypothyroidism: hazard ratio, 1.90; 95% CI, 1.14-3.19; high-normal TSH: hazard ratio, 1.36; 95% CI, 1.07-1.73) compared with the middle-normal TSH group. Cardiovascular disease mediated 14.3% and 5.9% of the associations of subclinical hypothyroidism and high-normal TSH with all-cause mortality, respectively, with the CVD mediation being most pronounced in women (7.5%-13.7% of the association) and participants aged 60 years and older (6.0%-14.8% of the association).

**Conclusions and Relevance:**

In this study, CVD mediated the associations of subclinical hypothyroidism and high-normal TSH concentrations with all-cause mortality in the US general population. Further studies are needed to examine the clinical benefit of thyroid hormone replacement therapy targeted to a middle-normal TSH concentration or active CVD screening for people with elevated TSH concentrations.

## Key Points

**Question:**

To what extent are subclinical hypothyroidism or high-normal serum thyrotropin (TSH) concentrations associated with mortality through cardiovascular disease among US adults?

**Findings:**

In this cohort study of 9020 US adults, higher TSH levels were associated with increased risk of all-cause mortality. Cardiovascular disease mediated 14.3% and 5.9% of the association of subclinical hypothyroidism and high-normal TSH concentrations with all-cause mortality, respectively.

**Meaning:**

This study found that cardiovascular disease mediated the association between subclinical hypothyroidism and all-cause mortality, indicating that investigations are needed to examine the clinical benefit of medical intervention for people with elevated TSH concentrations.

## Teaser

This cohort study estimates the extent to which subclinical hypothyroidism or high-normal thyrotropin (TSH) concentrations are associated with mortality through cardiovascular disease among US adults.

## Introduction

Subclinical hypothyroidism, or elevated thyrotropin (TSH) levels with normal levels of free thyroxine (FT_4_), is a common disorder affecting approximately 10% of the adult population.^1^ A previous review^2^ has reported cardiovascular abnormalities such as left ventricular dysfunction and impaired vascular relaxation among people with not only overt hypothyroidism but also subclinical hypothyroidism. Inadequate serum thyroid hormone produces impairment of cardiac function and may result in bradycardia, endothelial dysfunction, increased intima-media thickness, diastolic dysfunction, increased vascular resistance, and pericardial effusion.^3^ Other epidemiological studies^4,5,6,7^ have also found an association between subclinical hypothyroidism or high-normal TSH concentrations (ie, serum TSH concentrations at the upper end of the normal range) and dyslipidemia, increased insulin resistance, and hypertension—components of metabolic syndrome.

Despite these mechanisms and evidence about the potential burden of elevated TSH on cardiac metabolism, the potentially causal link between subclinical hypothyroidism, cardiovascular disease (CVD), and death remains unclear. Recent individual patient meta-analysis by the Thyroid Studies Collaboration^8,9,10,11^ did not find higher risk of CVD and all-cause mortality among adults with subclinical hypothyroidism compared with those with euthyroidism, which is supported by current clinical guidelines proposing that thyroid hormone replacement therapy should not be routinely offered for adults with subclinical hypothyroidism.^12,13,14^ Meanwhile, some recent studies^5,15,16^ have suggested that even high-normal TSH concentrations are associated with increased risk of CVD and mortality among the US general population or adults with chronic kidney disease, indicating the importance of considering variations in the normative range of TSH when assessing long-term adverse health outcomes.

Mediation analysis has been increasingly used in clinical research to help evaluate underlying mechanisms of diseases.^17^ This epidemiological method decomposes the total association of a risk factor with the outcome (eg, subclinical hypothyroidism or high-normal TSH concentrations with mortality) into a direct association (not through CVD) and an indirect association (through CVD).^18^ The distinction of direct and indirect associations helps us to consider the potential effectiveness of intervention strategies; if there are substantial indirect associations for subclinical hypothyroidism or high-normal TSH concentrations, active screening for CVD for patients with subclinical hypothyroidism may be helpful to mitigate the increased mortality risk in this group.

Based on the association between hypothyroidism and CVD, we used a mediation analysis to investigate the strength of any mediation from subclinical hypothyroidism or high-normal TSH concentrations to all-cause mortality via CVD in the general US population.

## Methods

### Data Sources and Study Population

The US National Health and Nutrition Examination Survey (NHANES) is a stratified, multistage probability sample of individuals selected at random from the general population through a complex statistical process. Available data include structured interview data and physical examination results, including urine and/or blood samples.^19^ The NHANES study protocols were approved by the National Center for Health Statistics institutional review board,^20^ and all participants provided informed written consent at enrollment. The present study is a secondary analysis of the existing data (NHANES) and, therefore, received exemption from the institutional review board at University of California, Los Angeles. Data were collected continuously but released in 2-year cycles. The present study includes data from 4 cycles of the continuous NHANES cohort (2001-2002, 2007-2008, 2009-2010, and 2011-2012) that collected data on serum FT_4_ and TSH concentrations. All participants were prospectively followed up for mortality through December 2015. Data were analyzed from January to August 2019. This study followed the Strengthening the Reporting of Observational Studies in Epidemiology (STROBE) reporting guideline.

There were 10 126 participants aged 20 years or older at enrollment for whom serum TSH and FT_4_ concentrations were available. We excluded participants who lacked data for education (10 participants), smoking (7 participants), or a death record (11 participants). We further excluded 189 participants who were pregnant or lactating at the time of blood draw and 508 individuals who reported use of medications that may alter serum thyroid function (amiodarone, thyroid hormone replacement, and/or antithyroidal drugs). A total of 381 participants with serum TSH concentrations below the lower limit of the normal range or abnormal serum FT_4_ concentrations were also excluded. The final analytical cohort included 9020 participants.

### Definition of Serum Thyroid Function

In the NHANES data sets, serum TSH from participants was measured with a microparticle enzyme immunoassay, and serum FT_4_ was measured with a 2-step enzyme immunoassay. The normal ranges for TSH and FT_4_ were defined as 0.34 to 5.60 mIU/L and 0.6 to 1.6 ng/dL (to convert FT_4_ to picomoles per liter, multiply by 12.871), respectively.^21,22^ Participants with serum TSH and FT_4_ concentrations within the normal range were considered to have euthyroid. Participants with serum TSH concentrations greater than the upper limit of the normal range and FT_4_ concentrations within the normal range were considered to have subclinical hypothyroidism.^4^ Given the potential U-curve association between TSH and mortality,^5^ we substratified participants with biochemically euthyroid into tertiles by serum TSH concentrations in our mediation analyses as follows: low-normal, 0.34 to 1.19 mIU/L; middle-normal, 1.20 to 1.95 mIU/L; and high-normal, 1.96 to 5.60 mIU/L.

### CVD and Other Covariates

Coronary artery diseases and nonfatal CVD events (congestive heart failure, angina, myocardial infarction, or stroke) were ascertained by self-report at baseline. Demographic variables included respondents’ age, sex, race/ethnicity, and education status. Smoking status; history of cancer, diabetes, hypertension, or dyslipidemia; and receipt of a statin prescription were self-reported. Weight and height were measured and used to calculate body mass index (BMI; calculated as weight in kilograms divided by height in meters squared). Serum creatinine measurements were performed according to the laboratory procedure manual for NHANES 2001 to 2002, 2007 to 2008, 2009 to 2010, and 2011 to 2012,^23^ from which an estimated glomerular filtration rate (measured in milliliters per minute per 1.73 meters squared) was calculated as previously described.^5,24^

### Outcome Ascertainment

The primary outcome was all-cause mortality. Mortality data were ascertained by the National Center for Health Statistics from death certificate information provided by the National Death Index after record matching by Social Security number, name, date of birth, race/ethnicity, sex, state of birth, and state of residence. Time to event was calculated from the day of TSH measurement to the end of follow-up or the death date.

### Statistical Analysis

The mean and standard deviation were calculated for continuous variables and the proportion was calculated for categorical variables in each category substratified by TSH concentrations. Multivariable Cox proportional hazards regression models adjusting for potential confounders were used to estimate hazard ratios of mortality for TSH concentration. We used restricted cubic spline models fitted for Cox proportional hazards models with 3 knots at the 10th, 50th, and 90th percentiles of TSH.^25^ Model 1 adjusted for age (continuous and square transformed), sex (men or women), race/ethnicity (non-Hispanic white, non-Hispanic black, Mexican American, or other race/ethnicity), education status (less than high school, high school or general education degree, or more than high school), and smoking (never, current, or former). Model 2 further adjusted for cancer history (yes or no) and estimated glomerular filtration rate (continuous) in addition to covariates in model 1. In our main analysis, we did not adjust for diabetes, hypertension, dyslipidemia, and BMI because they can be mediators on the pathway from thyroid function to CVD.

In mediation analyses, we aimed to quantify the degree to which CVD mediates the association between thyroid function (ie, low-normal TSH, high-normal TSH, and subclinical hypothyroidism; reference group was middle-normal TSH) and mortality adjusting for potential confounders included in model 2 (eFigure 1 in the Supplement). We used a marginal structural approach within the counterfactual framework.^18,26,27^ The mediated proportion was computed as the pure natural estimated indirect effect size divided by the estimated total effect size. We included cross-product terms of exposure and mediator in the model given the previous findings that underlying cardiovascular status can be a modifier of the association between hypothyroidism and the long-term adverse health outcomes.^28,29^ More detailed discussion and coding are described elsewhere.^26,27^

As the TSH distribution differs by sex and age,^30^ stratum-specific analyses were conducted to estimate the associations of TSH with mortality according to sex (men and women) and age (<60 and ≥60 years) using category-specific tertiles to define low-normal, middle-normal, and high-normal TSH in each analysis. In sensitivity analyses, we further adjusted for diabetes (yes or no), hypertension (yes or no), statin prescription (yes or no), and BMI (continuous and square transformed) in addition to covariates in model 2 given the possibility that they could be confounders (ie, affecting thyroid function) rather than mediators (ie, affected by thyroid function). We used statin prescription information as a marker of lipid disorders instead of a diagnosis of dyslipidemia because the latter had approximately 40% missing data. To assess the robustness of our findings, we also performed the main analysis using alternate definitions for normal range serum thyroid function: TSH, 0.4 to 4.3 mIU/L, and FT_4_, 0.6 to 1.6 ng/dL.^31^

Statistical analyses were conducted using Stata software version 15 (StataCorp). We selected appropriate sample weights to account for unequal probabilities of selecting NHANES participants, as well as nonresponse of those eligible and approached.^32^ We confirmed that there was no evidence for violation of the proportional hazards assumption for TSH concentrations and CVD using Schoenfeld residuals (*estat phtest* in Stata). Bias-corrected 95% confidence intervals were estimated by repeating the analysis on 1000 bootstrapped samples. All statistical tests were 2-sided and considered significant at *P* < .05.

## Results

Of 9020 participants, 4658 (51.6%) were men; the mean (SD) age was 49.4 (17.8) years. Participants with subclinical hypothyroidism and high-normal TSH were generally older, and serum TSH was higher in women, non-Hispanic white participants, those with less education, never smokers, and those with hypertension and CVD (Table 1).

**Table 1.** Baseline Characteristics of 9020 Patients According to Serum TSH Concentrations in the National Health and Nutrition Examination Survey, 2001 to 2002 and 2007 to 2012^a^

Characteristic	No. (%)			
	TSH Concentration			Subclinical Hypothyroidism
	Low-Normal	Middle-Normal	High-Normal	
Participants, No.	2970	2934	2951	165
TSH range, mIU/L	0.34-1.19	1.20-1.95	1.96-5.60	>5.60
Mean (SD)	0.86 (0.22)	1.55 (0.22)	2.87 (0.80)	8.64 (5.12)
Median (IQR)	0.89 (0.69-1.05)	1.54 (1.36-1.73)	2.65 (2.24-3.27)	6.95 (6.13-8.10)
Free thyroxine range, ng/dL	0.60-1.50	0.60-1.57	0.60-1.40	0.60-1.10
Mean (SD)	0.81 (0.13)	0.80 (0.12)	0.78 (0.12)	0.74 (0.11)
Median (IQR)	0.80 (0.70-0.90)	0.80 (0.70-0.90)	0.80 (0.70-0.87)	0.70 (0.68-0.80)
Age, mean (SD), y	46.2 (17.3)	48.9 (17.3)	52.7 (17.9)	57.1 (18.0)
Men	1547 (52.0)	1541 (52.5)	1491 (50.5)	79 (47.9)
Race/ethnicity				
Non-Hispanic white	1149 (38.6)	1325 (45.1)	1589 (53.8)	108 (65.5)
Non-Hispanic black	799 (26.9)	569 (19.4)	398 (13.5)	7 (4.2)
Mexican American	491 (16.5)	516 (17.6)	503 (17.0)	23 (13.9)
Other	536 (18.0)	525 (17.9)	464 (15.7)	27 (16.4)
Education status				
<9th grade	347 (11.7)	355 (12.1)	417 (14.1)	30 (18.2)
9th-11th grade	537 (18.1)	475 (16.2)	481 (16.3)	20 (12.1)
High school or General Educational Development	729 (24.5)	672 (22.9)	658 (22.3)	31 (18.8)
>High school	1362 (45.8)	1433 (48.8)	1398 (47.3)	84 (50.9)
Smoking				
Never	1492 (50.2)	1556 (53.0)	1646 (55.7)	93 (56.4)
Current	801 (26.9)	643 (21.9)	523 (17.7)	26 (15.8)
Former	682 (22.9)	736 (25.1)	785 (26.6)	46 (27.9)
Body mass index, mean (SD)^b^	28.0 (6.1)	28.9 (6.5)	29.4 (7.1)	28.3 (6.0)
Estimated glomerular filtration rate, mean (SD), mL/min/1.73 m^2^	97.5 (24.9)	95.0 (25.5)	90.9 (25.6)	83.9 (27.5)
Diabetes				
Yes	315 (10.8)	326 (11.3)	392 (13.6)	16 (9.7)
Missing or unknown	51 (1.7)	44 (1.5)	65 (2.2)	1 (0.6)
Hypertension				
Yes	907 (30.6)	979 (33.5)	1105 (37.5)	63 (38.2)
Missing or unknown	7 (0.2)	9 (0.3)	5 (0.2)	0
Statin prescription	303 (10.2)	356 (12.3)	438 (14.8)	21 (12.7)
Cancer history				
Yes	199 (6.7)	276 (9.4)	337 (11.4)	18 (10.9)
Missing or unknown	5 (0.2)	2 (0.1)	4 (0.1)	0
Cardiovascular disease				
Yes	255 (8.6)	275 (9.4)	365 (12.4)	22 (13.6)
Missing or unknown	20 (0.7)	10 (0.3)	20 (0.7)	3 (0.2)

Abbreviations: IQR, interquartile range; TSH, thyrotropin.

SI conversion factor: To convert free thyroxine to pmol/L, multiply by 12.871.

^a^ Euthyroidism was defined as serum TSH and free thyroxine levels within the reference range (TSH, 0.34-5.60 mIU/L; free thyroxine, 0.6-1.6 ng/dL). Subclinical hypothyroidism was defined as serum TSH level higher than upper limit of normal range and free thyroxine levels within the reference range.

^b^ Calculated as weight in kilograms divided by height in meters squared.

### TSH and Mortality

The median (interquartile range) duration of follow-up for mortality ascertainment was 7.3 (5.4-8.3) years, during which 435 deaths from all causes were identified. After adjusting for all potential confounders in model 2, restricted cubic splines showed a U-curved association between TSH and the risk of all-cause mortality (Figure). When we compared with the middle-normal TSH group, the hazard ratios for all-cause mortality were higher for the low-normal TSH group (hazard ratio, 1.36; 95% CI, 1.01-1.83), high-normal TSH group (hazard ratio, 1.36; 95% CI, 1.07-1.73), and subclinical hypothyroidism group (hazard ratio, 1.90; 95% CI, 1.14-3.19) (Table 2). Additional adjustment for indicators of metabolic disorders (ie, diabetes, hypertension, statin prescription, and BMI) did not alter these results (eFigure 2 and eTable 1 in the Supplement).

**Figure.**
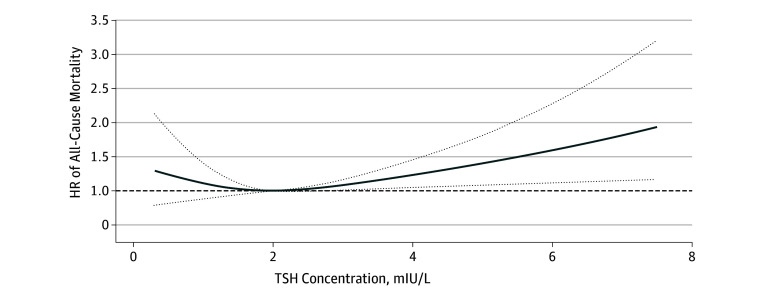
Association Between Serum Thyrotropin (TSH) Concentrations and All-Cause Mortality Using a Restricted Cubic Spline Regression Model in the National Health and Nutrition Examination Survey, 2001 to 2002 and 2007 to 2012, Followed up Through 2015 Results were adjusted for age, sex, race/ethnicity, education status, smoking, cancer history, and estimated glomerular filtration rate. Restricted cubic spline regression model was conducted with 3 knots at the 10th, 50th, and 90th percentiles of TSH. The dotted lines represent the 95% confidence intervals for the spline model (reference is 2.0 mIU/L). The range of TSH was restricted to 0.34 to 7.5 mIU/L because predictions greater than 7.5 mIU/L (95th percentile) are based on too few data points. HR indicates hazard ratio.

**Table 2.** Associations Between Serum Thyrotropin Concentrations and All-Cause Mortality in the National Health and Nutrition Examination Survey, 2001 to 2002 and 2007 to 2012, Followed up Through 2015

Thyrotropin Concentration	Deaths, No./Total No.	Adjusted HR (95% CI)		
		Age and Sex	Model 1^a^	Model 2^b^
Low-normal	124/2970	1.42 (1.06-1.90)	1.33 (0.99-1.79)	1.36 (1.01-1.83)
Middle-normal	117/2934	1 [Reference]	1 [Reference]	1 [Reference]
High-normal	173/2951	1.35 (1.06-1.71)	1.38 (1.09-1.76)	1.36 (1.07-1.73)
Subclinical hypothyroidism	21/165	1.90 (1.10-3.31)	1.96 (1.15-3.32)	1.90 (1.14-3.19)

Abbreviation: HR, hazard ratio.

^a^ Adjusted for age, sex, race/ethnicity, education status, and smoking.

^b^ Adjusted for cancer history and estimated glomerular filtration rate in addition to covariates in model 1.

### Mediation of TSH and Mortality Associations Through CVD

We estimated that CVD mediated 14.3% and 5.9% of the association of subclinical hypothyroidism and high-normal TSH with all-cause mortality, respectively (Table 3). We did not find any evidence for mediation through CVD for low-normal TSH levels and all-cause mortality. Additional adjustment for metabolic disorder indicators (ie, diabetes, hypertension, statin prescriptions, and BMI) or using a different definition of the normal range for TSH did not qualitatively alter these results (eTable 2 and eTable 3 in the Supplement).

**Table 3.** Estimated Direct and Indirect Effect Sizes of Serum Thyrotropin Concentrations With All-Cause Mortality Through Cardiovascular Disease in the National Health and Nutrition Examination Survey, 2001 to 2002 and 2007 to 2012, Followed up Through 2015^a^

Thyrotropin Concentration	Deaths, No./Total No.	Hazard Ratio (95%CI)			% Mediated^b^
		Total Effect	Direct Effect	Indirect Effect	
Low-normal	124/2970	1.32 (0.89-1.92)	1.31 (1.00-1.72)	1.01 (0.76-1.32)	4.2
Middle-normal	117/2934	1 [Reference]	1 [Reference]	1 [Reference]	1 [Reference]
High-normal	173/2951	1.38 (1.13-1.81)	1.36 (1.11-1.76)	1.02 (1.00-1.05)	5.9
Subclinical hypothyroidism	21/165	1.92 (1.12-2.77)	1.75 (0.99-2.60)	1.10 (0.97-1.30)	14.3

^a^ Hazard ratios were adjusted for age, sex, race/ethnicity, education status, smoking, cancer history, and estimated glomerular filtration rate. Estimated total effects were slightly different from Table 2 owing to a different statistical approach. A total of 1000 iterations were performed for bootstrapping to estimate 95% bias-corrected confidence interval.

^b^ The percentage mediated was calculated by log(estimated indirect effect)/log(estimated total effect).

In stratified analyses, we estimated that CVD mediated 7.5% to 13.7% of the associations of high-normal TSH or subclinical hypothyroidism with all-cause mortality in women, while we found no mediation or only a slight mediation through CVD in men (Table 4). We also estimated that CVD mediated 6.0% of the associations of high-normal TSH with all-cause mortality for participants aged 60 years or older (eTable 4 in the Supplement). A mediation through CVD was also estimated at 14.8% for subclinical hypothyroidism, but the 95% confidence interval of the estimated indirect effect size included the null, reflecting the smaller sample size. We did not find evidence for CVD mediation between low-normal TSH and all-cause mortality overall or stratified by sex and age.

**Table 4.** Estimated Direct and Indirect Effect Sizes of Serum Thyrotropin Concentrations on All-Cause Mortality Through Cardiovascular Disease Stratified by Sex in the National Health and Nutrition Examination Survey, 2001 to 2002 and 2007 to 2012, Followed up Through 2015^a^

Thyrotropin Concentration	Deaths, No./Total No.	Hazard Ratio (95% CI)			% Mediated^b^
		Total Effect	Direct Effect	Indirect Effect	
Men^c^					
Low-normal	79/1527	1.51 (0.88-2.39)	1.48 (1.02-2.31)	1.02 (0.72-1.53)	4.7
Middle-normal	63/1525	1 [Reference]	1 [Reference]	1 [Reference]	1 [Reference]
High normal	100/1521	1.42 (1.07-2.08)	1.41 (1.04-2.06)	1.01 (0.99-1.06)	2.4
Subclinical hypothyroidism	11/79	1.66 (0.85-2.65)	1.65 (0.81-2.83)	1.00 (0.86-1.20)	0.9
Women^d^					
Low-normal	43/1429	1.14 (0.59-2.05)	1.13 (0.72-1.65)	1.01 (0.64-1.63)	6.2
Middle-normal	53/1426	1 [Reference]	1 [Reference]	1 [Reference]	1 [Reference]
High normal	76/1427	1.43 (1.04-1.98)	1.39 (1.01-1.93)	1.03 (1.00-1.08)	7.5
Subclinical hypothyroidism	10/86	2.13 (0.91-3.74)	1.92 (0.76-3.47)	1.11 (0.93-1.64)	13.7

^a^ Adjusted for age, sex, race/ethnicity, education status, smoking, cancer history, and estimated glomerular filtration rate. A total of 1000 iterations were performed for bootstrapping to estimate 95% bias-corrected confidence interval.

^b^ Percentage mediated was calculated by log(estimated indirect effect)/log(estimated total effect).

^c^ For men, low-normal was defined as 0.34 to 1.19 mIU/L; middle-normal, 1.20 to 1.93 mIU/L; and high-normal, 1.94 to 5.60 mIU/L.

^d^ For women, low-normal was defined as 0.34 to 1.19 mIU/L; middle-normal, 1.20 to 1.97 mIU/L; and high-normal, 1.98 to 5.60 mIU/L.

## Discussion

Using mediation analysis, we found that CVD mediated 14.3% of the associations of subclinical hypothyroidism with all-cause mortality and 5.9% of the associations of high-normal TSH with all-cause mortality among a representative sample of the US general adult population. The mediation of CVD was observed particularly among women and participants aged 60 years and older when stratified by sex and age. While low-normal TSH was associated with an increased risk of all-cause mortality, we did not find evidence of CVD mediation for this group.

To our knowledge, this is the first study to quantify the extent to which CVD mediates the association of serum thyroid function on all-cause mortality in the US general population. Current clinical guidelines from the American Thyroid Association^14^ and European Thyroid Association^13^ do not recommend treating subclinical hypothyroidism when TSH is 10 mIU/L or less (and without symptoms in younger individuals). This recommendation has been followed by 2 recent comprehensive reviews^1,12^ that summarized the evidence arguing against clinical benefits of thyroid hormone replacement therapy toward quality of life or hypothyroid symptoms among adults, especially elderly individuals, with subclinical hypothyroidism. However, it remains unclear whether thyroid hormone replacement therapy is effective in mitigating several long-term adverse outcomes, such as CVD and death, in the general population with subclinical hypothyroidism.^12^ A previous meta-analysis^8^ reported that subclinical hypothyroidism is associated with higher CVD risk, and thus treatment with levothyroxine is recommended when serum TSH concentrations reach 10 mIU/L or greater.^4,31^ Our findings of increased risk of all-cause mortality among participants with subclinical hypothyroidism (defined by TSH >5.6 mIU/L) mediated by CVD, compared with those with middle-normal TSH concentrations, suggest that higher TSH level may be a risk factor for CVD and also mortality, even when TSH levels are only modestly elevated. This was further supported by our results based on restricted cubic spline curves, which allowed for flexibility examining in the association between continuous TSH concentrations and the risk of mortality.

In the present study, we found a U-curved association between serum TSH concentrations and risk of all-cause mortality, ie, even within normative serum TSH and FT_4_ concentrations. Moreover, CVD mediated the association between high-normal TSH and all-cause mortality. While the observed estimated indirect effect sizes were small for high-normal TSH, the impact of these associations should be considered, given that participants with high-normal TSH compose almost one-third of the total population (by definition) in the present study. Results from previous studies^5,33,34,35,36^ that examined associations of serum thyroid function within the normal range and various long-term adverse outcomes have been inconsistent across different populations. Rhee and colleagues^15,16^ have reported an association between high-normal TSH concentrations and mortality among people with chronic kidney disease, including those who are receiving dialysis. Our findings provide additional support for the importance of considering variations in the normative range of thyroid hormone levels (statistically defined as between percentiles 2.5 and 97.5 in a healthy population) when assessing long-term mortality risks in the general US population.

There are several potential mechanisms that may explain the associations of subclinical hypothyroidism and high-normal TSH (ie, low-normal thyroid function) with death mediated through CVD. The thyroid hormone is one of the key regulators of cardiac function and cardiovascular hemodynamics; therefore, inadequate thyroid hormone levels impair the relaxation of vascular smooth muscle cells and decrease cardiac contractility by regulating calcium uptake and the expression of several contractile proteins in cardiomyocytes.^37,38^ Low thyroid hormone levels also increase systemic vascular resistance and induce endothelial dysfunction by reducing nitric oxide availability.^37,38,39^ Previous studies,^5,6,7,40^ including those using NHANES data,^5,7^ reported that low thyroid function even within the normal range is associated with metabolic syndrome owing to an increase in insulin resistance and might contribute to the progression of atherosclerosis and CVD risk.^41^ While the exact underlying mechanisms are still unclear, these biological mechanisms support our finding that CVD partially mediates the association of higher TSH concentrations with death.

In our stratified analyses, we found that mediation through CVD was most pronounced in women. In general, it is well established that women are at an increased risk of progression from subclinical to overt hypothyroidism.^4^ A previous study from Norway^42^ also found an association between high-normal TSH concentrations and CVD in women but not in men, consistent with our results. We also found that high-normal TSH was associated with increased risk of all-cause mortality both in participants younger than 60 years and in those 60 years or older; however, we had insufficient power to estimate statistically significant effect sizes in younger participants, which will require larger studies with longer follow-up periods. The mediation of CVD on the association between higher TSH concentrations and all-cause mortality was observed only in older participants. Although thyroid hormone therapy should not be routinely offered to patients with subclinical hypothyroidism, our findings indicate the importance of further investigations regarding clinical effectiveness of medical interventions (such as a CVD prevention program) for specific populations with subclinical hypothyroidism or even low-normal thyroid function.

We also observed an association between low-normal TSH concentrations (ie, high-normal thyroid function) and all-cause mortality, especially in men and in the younger population. However, we did not find any evidence for a mediation through CVD. Previous studies^10,34,36,43^ have found an increased risk of CVD and mortality among people with high-normal thyroid function partially mediated by anticoagulant factors. Elevated thyroid hormone levels have been reported to negatively affect quality of life and cognitive function as well as the cardiovascular system in young and middle-aged populations.^4^ Moreover, Collet et al^10^ reported that the mortality risk at lower TSH concentrations was slightly greater in men than in women. Additional studies are needed to identify factors responsible for the association of lower TSH concentrations with death and to corroborate the observed heterogeneity by sex and age.

### Strengths and Limitations

A major strength of our study includes estimation of a potential pathway from serum TSH concentrations to mortality through CVD in a large, nationally representative sample of the US general population with individualized linkage to the national mortality database with rigorous capture of death events. Moreover, we used both serum TSH and FT_4_ to define subclinical hypothyroidism, which has not been implemented in previous studies using NHANES III owing to the lack of FT_4_ data (ie, only total thyroxine data are available in NHANES III).^5,29,44^

However, our study also has several limitations. First, it was not possible to consider any trends or changes in thyroid function over follow-up. Thus, the present study does not provide any information regarding the importance of treatment with thyroid hormone replacement if high TSH levels subsequently triggered medication use in NHANES participants. Second, while it is unlikely that CVD affected serum TSH concentrations, the exposure-mediator association is not well defined temporarily, and we need to consider the possibility of reverse causation (ie, that the participants’ thyroid status prior to CVD was different from their thyroid status at the time of study entry). Third, as CVD and other medical histories were self-reported, the mediator and confounders might have been misclassified. Even though these are all likely causing nondifferential misclassifications, the bias this may generate is not always toward the null in mediation analysis.^45^ Fourth, our mediation analysis was based on the assumption that there were no other unmeasured confounders and no confounding between the mediator and outcome as affected by exposure.^45^ Therefore, we cannot rule out the risk of bias due to unmeasured confounders. While metabolic disorders such as diabetes, hypertension, dyslipidemia, and obesity can be confounders between mediator and outcome affected by exposure, the consistency of our results between the main model and the model additionally adjusted for these metabolic disorders indicates that any potential bias due to a violation of this assumption might be small. In addition, our data did not capture all of the CVD events during the follow-up period; therefore, effect size of the CVD mediation might be underestimated. Future investigation with larger sample sizes or additional outcome events and longitudinal measures of TSH levels and CVD events are warranted to overcome these limitations, to replicate and validate our findings, and to estimate the overall CVD mediation of the association between thyroid function and mortality.

## Conclusions

In conclusion, using a nationally representative database of US adults, this study found that CVD mediated the associations of subclinical hypothyroidism and high-normal TSH concentrations with all-cause mortality, especially in women and possibly in older individuals. We did not find evidence of CVD mediation on the pathway from low-normal TSH concentrations to all-cause mortality. Further studies are needed to examine the clinical benefit of thyroid hormone replacement therapy targeted to a mid-normal TSH concentration or active CVD screening for people with elevated TSH concentrations.

## Notes

Supplement. **eFigure 1.** Causal Diagram Under Investigation **eFigure 2.** Association Between Serum TSH Concentrations and All-Cause Mortality Additionally Adjusted for Comorbidities Using a Restricted Cubic Spline Regression Model in NHANES 2001-2002 and 2007-2012 Followed Through 2015 **eTable 1.** Associations Between Serum TSH Concentrations and All-Cause Mortality Additionally Adjusted for Comorbidities in NHANES 2001-2002 and 2007-2012 Followed Through 2015 **eTable 2.** Direct and Indirect Effects (Hazard Ratio Scale [95%CI]) of Serum TSH Concentrations on All-Cause Mortality Through Cardiovascular Disease Additionally Adjusted for Comorbidities in NHANES 2001-2002 and NHANES 2007-2012 Followed Through 2015 **eTable 3.** Direct and Indirect Associations (Hazard Ratio Scale [95%CI]) of Serum TSH Concentrations on All-Cause Mortality Through Cardiovascular Disease Using Different Cut-off for TSH (0.4-4.3 mIU/L as Normal Range) in NHANES 2001-2002 and NHANES 2007-2012 Followed Through 2015 **eTable 4.** Direct and Indirect Associations (Hazard Ratio Scale [95%CI]) of Serum TSH Concentrations on All-Cause Mortality Through Cardiovascular Disease Stratified by Age in NHANES 2001-2002 and 2007-2012 Followed Through 2015
