# Supplementary material for: Association of Subclinical Hypothyroidism and Cardiovascular Disease With Mortality
Source: JAMA Netw Open. 2020 Feb 7;3(2):e1920745. doi: 10.1001/jamanetworkopen.2019.20745 (PMC12064075; doi:10.1001/jamanetworkopen.2019.20745)
Supplement: Supplement. — eFigure 1. Causal Diagram Under Investigation eFigure 2. Association Between Serum TSH Concentrations and All-Cause Mortality Additionally Adjusted for Comorbidities Using a Restricted Cubic Spline Regression Model in NHANES 2001-2002 and 2007-2012 Followed Through 2015 eTable 1. Associations Between Serum TSH Concentrations and All-Cause Mortality Additionally Adjusted for Comorbidities in NHANES 2001-2002 and 2007-2012 Followed Through 2015 eTable 2. Direct and Indirect Effects (Hazard Ratio Scale [95%CI]) of Serum TSH Concentrations on All-Cause Mortality Through Cardiovascular Disease Additionally Adjusted for Comorbidities in NHANES 2001-2002 and NHANES 2007-2012 Followed Through 2015 eTable 3. Direct and Indirect Associations (Hazard Ratio Scale [95%CI]) of Serum TSH Concentrations on All-Cause Mortality Through Cardiovascular Disease Using Different Cut-off for TSH (0.4-4.3 mIU/L as Normal Range) in NHANES 2001-2002 and NHANES 2007-2012 Followed Through 2015 eTable 4. Direct and Indirect Associations (Hazard Ratio Scale [95%CI]) of Serum TSH Concentrations on All-Cause Mortality Through Cardiovascular Disease Stratified by Age in NHANES 2001-2002 and 2007-2012 Followed Through 2015 [file jamanetwopen-e1920745-s001.pdf]

Supplementary Online Content

Inoue K, Ritz B, Brent GA, Ebrahimi R, Rhee CM, Leung AM. Association of subclinical hypothyroidism and cardiovascular disease with mortality. *JAMA Netw Open*. 2020;3(2):e1920745.
doi:10.1001/jamanetworkopen.2019.20745

eFigure 1. Causal Diagram Under Investigation
eFigure 2. Association Between Serum TSH Concentrations and All-Cause Mortality Additionally Adjusted for Comorbidities Using a Restricted Cubic Spline Regression Model in NHANES 2001-2002 and 2007-2012 Followed Through 2015
eTable 1. Associations Between Serum TSH Concentrations and All-Cause Mortality Additionally Adjusted for Comorbidities in NHANES 2001-2002 and 2007-2012 Followed Through 2015
eTable 2. Direct and Indirect Associations (Hazard Ratio Scale [95%CI]) of Serum TSH Concentrations on All-Cause Mortality Through Cardiovascular Disease Additionally Adjusted for Comorbidities in NHANES 2001-2002 and NHANES 2007-2012 Followed Through 2015
eTable 3. Direct and Indirect Associations (Hazard Ratio Scale [95%CI]) of Serum TSH Concentrations on All-Cause Mortality Through Cardiovascular Disease Using Different Cut-off for TSH (0.4-4.3 mIU/L as Normal Range) in NHANES 2001-2002 and NHANES 2007-2012 Followed Through 2015
eTable 4. Direct and Indirect Associations (Hazard Ratio Scale [95%CI]) of Serum TSH Concentrations on All-Cause Mortality Through Cardiovascular Disease Stratified by Age in NHANES 2001-2002 and 2007-2012 Followed Through 2015

This supplementary material has been provided by the authors to give readers additional information about their work.

© 2020 Inoue K et al. *JAMA Network Open.*

eFigure 1. Causal diagram under investigation

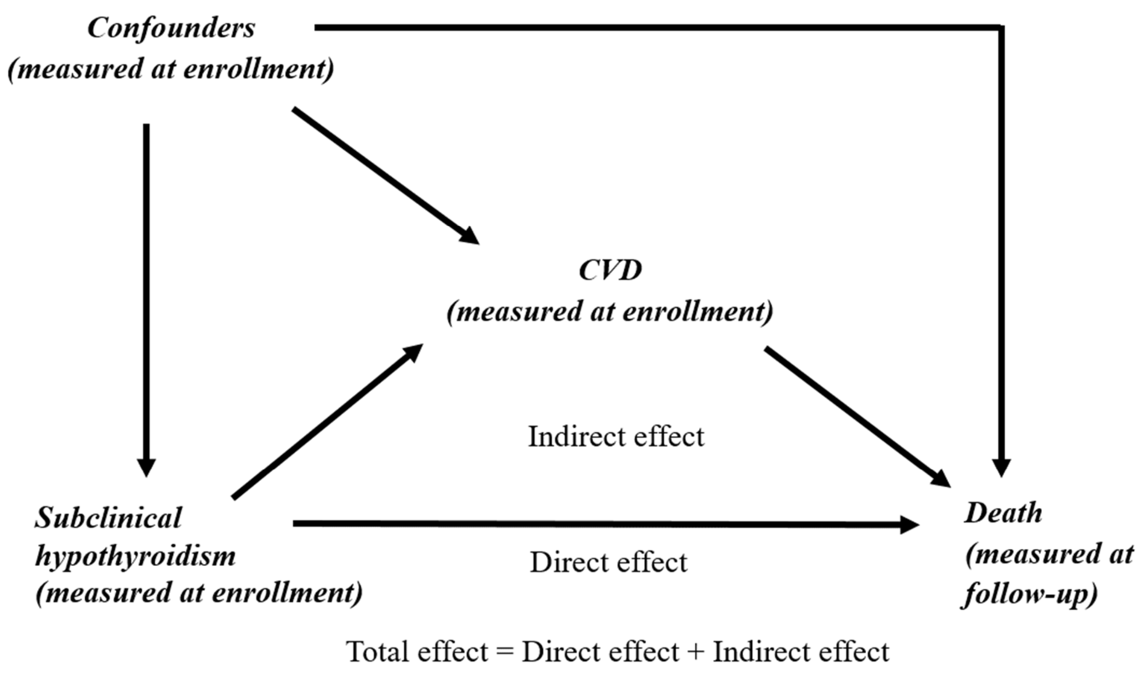

Confounders in our main Model (Model 2): age, sex, race/ethnicity, education status, smoking, previous cancer history, and eGFR. We also adjusted for diabetes, hypertension, statin prescription, and BMI in addition to covariates in Model 2 in the sensitivity analysis.

© 2020 Inoue K et al. *JAMA Network Open.*

eFigure 2. Association between serum TSH concentrations and all-cause mortality additionally adjusted for comorbidities using a restricted cubic spline regression model in NHANES 2001-2002 and 2007-2012 followed through 2015.

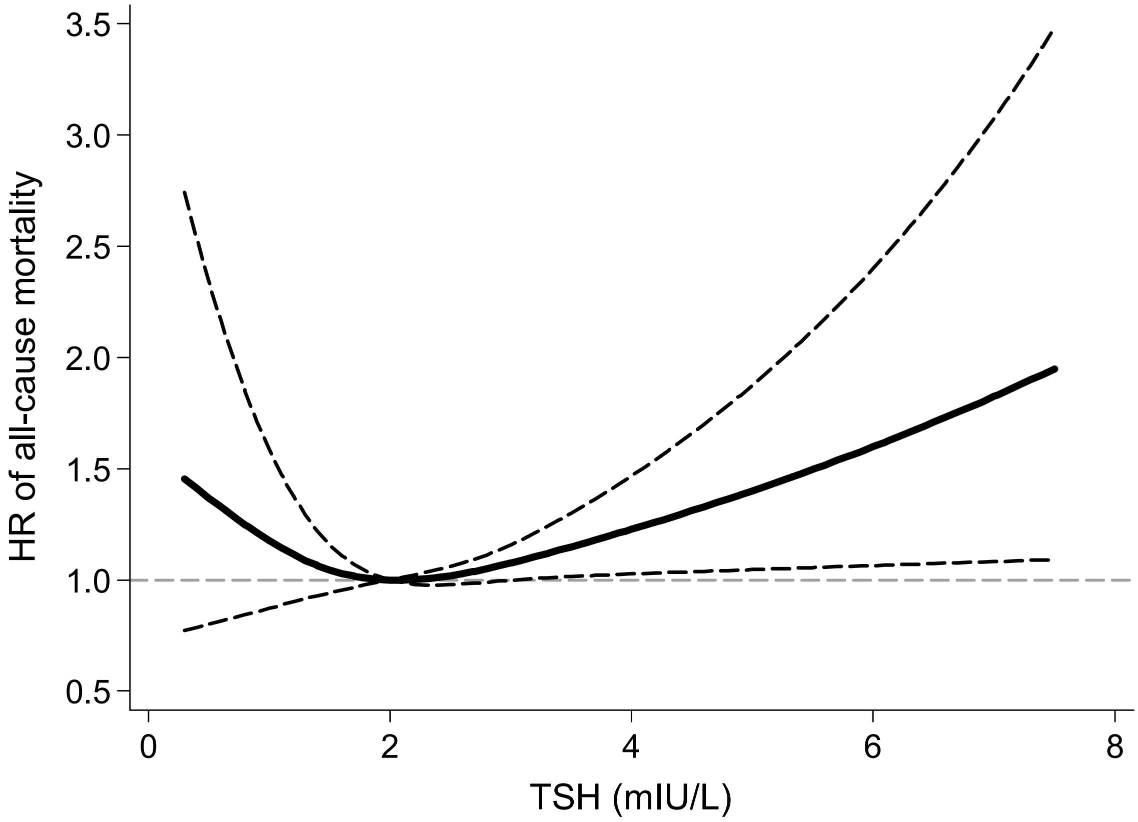

Adjusted for age, sex, race/ethnicity, education status, smoking, previous cancer history, eGFR, diabetes, hypertension, statin prescription, and BMI. Restricted cubic spline regression model was conducted with three knots at 10th, 50th, and 90th percentile of TSH (mIU/L). The dashed lines represent the 95% CIs for the spline model (reference is 2.0 mIU/L). The range of TSH was restricted to 0.34–7.5 mIU/L because predictions >7.5 mIU/L (95th percentile) are based on too few data point

© 2020 Inoue K et al. *JAMA Network Open.*

eTable 1. Associations between serum TSH concentrations and all-cause mortality additionally adjusted for comorbidities in NHANES 2001-2002 and 2007-2012 followed through 2015.

All-cause mortality	Event N/ Total N	Adjusted HR (95% CI) [a]
**Low-normal TSH**	107/2865	1.30 (0.92-1.84)
**Middle-normal TSH**	105/2836	Ref
**High-normal TSH**	145/2818	1.20 (0.90-1.61)
**Subclinical hypothyroidism**	18/160	1.80 (1.02-3.19)

[a] HR adjusted for age, sex, race/ethnicity, education status, smoking, previous cancer history, eGFR, diabetes, hypertension, statin prescription, and BMI. The results did not change when we additionally adjusted for systolic blood pressure, HbA1c, cholesterol levels, antihypertensive medication use, and antidiabetic medication use.

© 2020 Inoue K et al. *JAMA Network Open.*

eTable 2. Direct and indirect Associations (hazard ratio scale [95%CI]) of serum TSH concentrations on all-cause mortality through cardiovascular disease additionally adjusted for comorbidities in NHANES 2001-2002 and NHANES 2007-2012 followed through 2015. [a,b]

	Event N/ Total N	Total effect (TE)	Direct effect (DE)	Indirect effect (IE)	%mediated [c]
**Low-normal TSH**	107/2865	1.28 (0.86-1.92)	1.27 (0.95-1.65)	1.00 (0.76-1.35)	1.7%
**Middle-normal TSH**	105/2836	Ref	Ref	Ref	Ref
**High-normal TSH**	145/2818	1.22 (0.97-1.59)	1.20 (0.96-1.57)	1.01 (1.00-1.04)	5.8%
**Subclinical hypothyroidism**	18/160	1.84 (1.13-2.75)	1.61 (0.92-2.58)	1.15 (0.97-1.59)	23.1%

[a] HR adjusted for age, sex, race/ethnicity, education status, smoking, previous cancer history, eGFR, diabetes, hypertension, statin prescription, and BMI.

[b] 1000 iterations were performed for bootstrapping to estimate 95% bias-corrected confidence interval.

[c] %mediated was calculated by log(IE)/log(TE).

© 2020 Inoue K et al. *JAMA Network Open.*

eTable 3. Direct and indirect Associations (hazard ratio scale [95%CI]) of serum TSH concentrations on all-cause mortality through cardiovascular disease using different cut-off for TSH (0.4-4.3 mIU/L as normal range) in NHANES 2001-2002 and NHANES 2007-2012 followed through 2015 [a,b]

	Event N/ Total N	Total effect (TE)	Direct effect (DE)	Indirect effect (IE)	%mediated [c]
**Low-normal TSH**	118/2854	1.34 (0.89-1.96)	1.32 (1.02-1.70)	1.02 (0.76-1.34)	5.8%
**Middle-normal TSH**	113/2856	Ref	Ref	Ref	Ref
**High-normal TSH**	166/2851	1.43 (1.15-1.84)	1.41 (1.11-1.78)	1.02 (1.00-1.05)	5.2%
**Subclinical hypothyroidism**	35/354	1.36 (0.91-1.90)	1.33 (0.88-1.85)	1.02 (0.93-1.13)	5.6%

Low-normal, 0.40–1.19 mIU/L; middle-normal, 1.20–1.91 mIU/L; and high-normal, 1.92–4.30 mIU/L.

[a] HR adjusted for age, sex, race/ethnicity, education status, smoking, previous cancer history, and eGFR.

[b] 1000 iterations were performed for bootstrapping to estimate 95% bias-corrected confidence interval.

[c] %mediated was calculated by log(IE)/log(TE).

© 2020 Inoue K et al. *JAMA Network Open.*

eTable 4. Direct and indirect Associations (hazard ratio scale [95%CI]) of serum TSH concentrations on all-cause mortality through cardiovascular disease stratified by age in NHANES 2001-2002 and 2007-2012 followed through 2015.[a,b]

**Age ≥60**	Event N/ Total N	Total effect (TE)	Direct effect (DE)	Indirect effect (IE)	%mediated[c]
**Low-normal TSH**	110/970	1.16 (0.79-1.72)	1.15 (0.87-1.47)	1.01 (0.76-1.32)	4.6%
**Middle-normal TSH**	95/969	Ref	Ref	Ref	Ref
**High normal TSH**	120/969	1.31 (1.03-1.70)	1.29 (1.01-1.68)	1.02 (1.00-1.05)	6.0%
**Subclinical hypothyroidism**	21/82	2.15 (1.35-3.35)	1.92 (1.09-2.84)	1.12 (0.94-1.44)	14.8%
**Age <60**	Event N/ Total N	Total effect (TE)	Direct effect (DE)	Indirect effect (IE)	%mediated[c]
**Low-normal TSH**	32/1996	1.56 (0.72-3.26)	1.56 (0.91-2.71)	1.00 (0.54-1.67)	0.0%
**Middle-normal TSH**	25/1970	Ref	Ref	Ref	Ref
**High normal TSH**	32/1981	1.56 (0.99-2.61)	1.58 (1.01-2.61)	0.99 (0.89-1.04)	-3.1%
**Subclinical hypothyroidism**	0/80	NA[d]			

Age ≥60: Low-normal, 0.34–1.34 mIU/L; middle-normal, 1.35–2.17 mIU/L; and high-normal, 2.18–5.60 mIU/L.

Age <60: Low-normal, 0.34–1.15 mIU/L; middle-normal, 1.16–1.84 mIU/L; and high-normal, 1.85–5.60 mIU/L.

[a] Adjusted for age, sex, race/ethnicity, education status, smoking, previous cancer history, and eGFR.

[b] 1000 iterations were performed for bootstrapping to estimate 95% bias-corrected confidence interval.

[c] %mediated was calculated by log(IE)/log(TE).

[d] The effect could not be calculated due to sparse data.

© 2020 Inoue K et al. *JAMA Network Open.*
